# A comparative clinical study of PF-06410293, a candidate adalimumab biosimilar, and adalimumab reference product (Humira®) in the treatment of active rheumatoid arthritis

**DOI:** 10.1186/s13075-018-1676-y

**Published:** 2018-08-15

**Authors:** Roy M. Fleischmann, Rieke Alten, Margarita Pileckyte, Kasia Lobello, Steven Y. Hua, Carol Cronenberger, Daniel Alvarez, Amy E. Bock, K. Lea Sewell

**Affiliations:** 1 Southwestern Medical Center, Metroplex Clinical Research Center, University of Texas, 8144 Walnut Hill Lane, Suite 810, Dallas, TX 75231 USA; 2 Schlosspark-Klinik, University Medicine Berlin, Heubnerweg 2, Berlin, 14059 Germany; 3 Department of Rheumatology, Hospital of Lithuanian University of Health Sciences, Eiveniu str.2, Kaunas, LT 50161 Lithuania; 4 Pfizer Inc., 500 Arcola Road Collegeville, Collegeville, PA 19426 USA; 5 Pfizer Inc., 10777 Science Center Drive, CB1/2103, San Diego, CA 92121 USA; 6 Pfizer Inc., 300 Technology Square, Cambridge, MA 02139 USA

**Keywords:** Rheumatoid arthritis, Adalimumab, Biosimilar, Comparative clinical study

## Abstract

**Background:**

This double-blind, randomized, 78-week study evaluated the efficacy, safety, immunogenicity, pharmacokinetics, and pharmacodynamics of PF-06410293, a candidate adalimumab biosimilar, versus adalimumab reference product (Humira^®^) sourced from the EU (adalimumab-EU) in biologic-naïve patients with active rheumatoid arthritis (RA) despite methotrexate (MTX) (10–25 mg/week). We report results for the first 26 weeks of treatment.

**Methods:**

Patients with active RA (*N* = 597) were randomly assigned (1:1) to PF-06410293 or adalimumab-EU, while continuing with MTX treatment. The primary endpoint was American College of Rheumatology 20% improvement (ACR20) at week 12. Therapeutic equivalence was concluded if the two-sided 95% confidence interval (CI) for the ACR20 difference between the two arms was entirely contained within the symmetric equivalence margin (±14%). Additionally, a two-sided 90% CI was calculated by using an asymmetric equivalence margin (−12%, 15%). Secondary efficacy endpoints to week 26 included ACR20/50/70, change from baseline Disease Activity Score based on high-sensitivity C-reactive protein [DAS28–4(CRP)], European League Against Rheumatism (EULAR) response, DAS28–4(CRP) of less than 2.6, and ACR/EULAR remission. QuantiFERON-TB testing was performed at screening and week 26.

**Results:**

Patients (78.7% of whom were female and whose mean age was 52.5 years) had a mean baseline RA duration of 6.8 years. The mean baseline DAS28–4(CRP) values were 5.9 (PF-06410293) and 6.1 (adalimumab-EU). The observed week-12 ACR20 values were 68.7% (PF-06410293) and 72.7% (adalimumab-EU) in the intention-to-treat population. With non-responder imputation, the treatment difference in week-12 ACR20 was −2.98% and corresponding CIs—95% CI (−10.38%, 4.44%) and 90% CI (−9.25%, 3.28%)—were entirely contained within the equivalence margins (symmetric and asymmetric, respectively). The secondary efficacy endpoints were similar between arms. Over 26 weeks, injection-site reactions occurred in 1.7% versus 2.0%, hypersensitivity events in 4.4% versus 8.4%, pneumonia in 0.7% versus 2.0%, and opportunistic infections in 2.4% versus 1.7% in the PF-06410293 and adalimumab-EU arms, respectively. One death due to myocardial infarction occurred (adalimumab-EU arm). Rates of anti-drug antibody incidence were 44.4% (PF-06410293) and 50.5% (adalimumab-EU).

**Conclusions:**

The study results demonstrate that efficacy, safety, and immunogenicity of PF-06410293 and adalimumab-EU were similar during the first 26 weeks of treatment in patients with active RA on background MTX.

**Trial registration:**

ClinicalTrials.gov Identifier: NCT02480153. First posted on June 24, 2015; EU Clinical Trials Register EudraCT number: 2014-000352-29. Start date: October 27, 2014.

**Electronic supplementary material:**

The online version of this article (10.1186/s13075-018-1676-y) contains supplementary material, which is available to authorized users.

## Background

The introduction of biologic disease-modifying anti-rheumatic drugs (bDMARDs) has been a major advance in the treatment of patients with rheumatoid arthritis (RA), providing an important addition to the previously available therapy options [1]. Adalimumab, a recombinant fully human immunoglobulin G1 monoclonal antibody, inhibits the interaction of tumor necrosis factor (TNF) with surface TNF receptors by specifically binding to TNF-α and has been shown to reduce clinical symptoms and inhibit radiographic progression in patients with RA [2–4]. Adalimumab is approved for multiple indications in addition to RA [5, 6].

The US Food and Drug Administration (FDA) defines a biosimilar as “a biopharmaceutical that is highly similar to an already licensed biologic product (the reference product), notwithstanding minor differences in clinically inactive components, and for which there are no clinically meaningful differences in purity, potency, and safety between the two products” [7]. The European Medicines Agency requires that a biosimilar show “similarity to the reference biologic with respect to quality, biologic activity, safety, and efficacy” [8]. Biosimilars may expand patient access to bDMARDs because of potentially lower drug prices as a result of price competition within the product market, resulting in savings for health-care systems and patients [9–11].

PF-06410293 is in development as a candidate adalimumab biosimilar. Peptide mapping data demonstrate that PF-06410293 has a primary amino acid sequence identical to that of adalimumab reference product and is similar in comparative analytical, functional, and binding assessments [12]. Pharmacokinetic (PK) similarity was demonstrated following single-dose administration of PF-06410293 and adalimumab to healthy volunteers (Pfizer unpublished observation) [13]. The current comparative clinical study compared the efficacy, safety, immunogenicity, PK, and pharmacodynamics (PD) of PF-06410293 with adalimumab reference product (Humira^®^) sourced from the EU (adalimumab-EU) in patients with active RA and an inadequate response to methotrexate (MTX).

## Methods

### Study population

Patients with active RA and an inadequate response to MTX represent a sensitive and appropriate population for biosimilar comparability trials. Adults (at least 18 years old) with a diagnosis of active RA at least 4 months, based on the 2010 American College of Rheumatology/European League Against Rheumatism (ACR/EULAR) criteria [14], were eligible for inclusion. Active RA was defined as at least six tender and at least six swollen joints (at screening and baseline) with a high-sensitivity C-reactive protein (hs-CRP) of at least 8 mg/L at screening (Additional file 1).

Patients were ineligible if they met any of the following criteria: prior treatment with adalimumab, lymphocyte-depleting therapy, or more than two doses of one biologic therapy; inadequate washout of any second DMARD, pregnancy or breastfeeding, clinically significant laboratory abnormalities, current infection, congestive heart failure (New York Heart Association grade 3/4), untreated or inadequately treated latent or active tuberculosis (TB), malignancy within the previous 5 years, or a positive test for human immunodeficiency virus or hepatitis B or C virus (Additional file 2).

### Study design and treatments

**Fig. 1:**
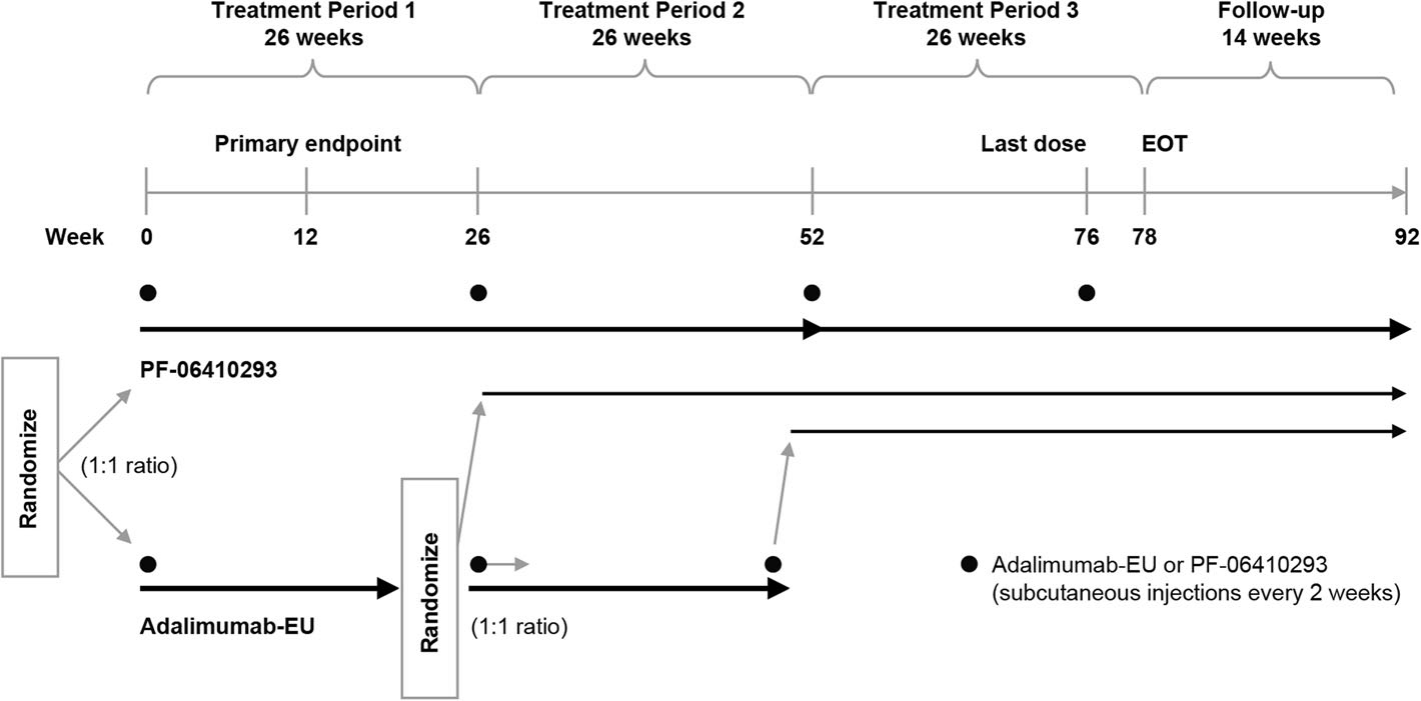
Study design. Abbreviations: *Adalimumab-EU* adalimumab sourced from the European Union, *EOT* end of treatment

This was a multinational, two-arm, double-blind, randomized, comparative clinical study in patients with active RA and was conducted at 173 centers in Australia, Brazil, Bulgaria, Colombia, the Czech Republic, Estonia, Georgia, Germany, Hungary, Japan, Lithuania, Mexico, New Zealand, Peru, Poland, the Republic of Korea, Serbia, South Africa, Spain, Taiwan, Ukraine, the Russian Federation, UK, and the US. Patients were randomly assigned (1:1) on day 1 (stratified by geographic region) to receive either PF-06410293 or adalimumab-EU. There were three 26-week treatment periods and a 16-week follow-up after last dose of study drug (Fig. 1). Prior to dosing at week 26, patients in the adalimumab-EU arm were blindly re-randomized (1:1) to continue on adalimumab-EU or switch to PF-06410293. At week 52, all patients remaining on adalimumab-EU were switched to PF-06410293 for open-label treatment during the third treatment period. Herein, we report data from the first 26 weeks of the study. The number of tender (68) and swollen (66) joints was determined by an independent blinded joint assessor.

PF-06410293 or adalimumab-EU was administered as a subcutaneous injection (40 mg every other week using a prefilled syringe) in addition to a stable background dose of oral or intramuscular MTX (10–25 mg/week) and oral folic/folinic acid; lower doses of MTX (6 mg/week) were allowed if indicated in local guidance or standards of care. Patients could receive concomitant low-dose oral corticosteroids (≤10 mg prednisone or equivalent per day), one non-steroidal anti-inflammatory drug, and non-opioid or specific opioid analgesics or both. Treatment could be delayed by up to 24 h prior to the next injection for illness or scheduling issues. Dosing could be temporarily held at the discretion of the investigator for an adverse event (AE) and resumed after the AE resolved, unless the patient missed three sequential injections.

### Primary study endpoint

The primary efficacy endpoint was the proportion of patients achieving an ACR20 response [15] at week 12. Week 12 is considered the beginning of the plateau of the time-response curve for ACR20 and, as such, is a more sensitive time point for the assessment of rapidity of response in biosimilar comparability trials in RA as suggested by regulatory authorities. Therefore, this trial evaluated the primary endpoint at week 12 rather than week 26, as used in the historical registration trials for adalimumab in patients with RA.

### Secondary endpoints and assessments

Secondary efficacy endpoints through week 26 included ACR20 (at time points in addition to week 12), ACR50, ACR70, change from baseline in Disease Activity Score 28 joints: four components based on hs-CRP [DAS28–4(CRP)], EULAR response, DAS28–4(CRP) of less than 2.6, ACR/EULAR remission, and change from baseline in individual ACR components, including Health Assessment Questionnaire Disability Index (HAQ-DI). The sponsor selected DAS28–4(CRP) rather than DAS28–4(erythrocyte sedimentation rate [ESR]) to determine clinical response, as CRP is performed in a central laboratory. A cutoff of less than 2.6 was used to define DAS28(CRP) “remission” and not more than 3.2 as “low disease activity” rather than the lower numbers that have been shown to best correlate with the DAS28(ESR) formula [16].

Safety endpoints included type, incidence, severity, timing, seriousness, and investigator-determined relatedness of AEs—using the National Cancer Institute Common Terminology Criteria for Adverse Events (Version 4.03)—and laboratory abnormalities (Covance, Indianapolis, IN, USA). Safety evaluations during study treatment included physical examinations, electrocardiograms, and QuantiFERON-TB Gold testing (at screening and week 26).

Prespecified treatment-emergent adverse events (TEAEs) of special interest were injection-site reactions (ISRs), opportunistic infections (defined for this study to include zoster, cytomegalovirus, latent/active TB, atypical mycobacteria, systemic fungal infections and oral thrush, pneumocystis, legionella, salmonellosis, shigellosis, vibrio, and other infections), and anaphylaxis/angioedema/urticaria. Additional prespecified TEAE categories of interest included blood and lymphatic events, cardiovascular events, demyelinating conditions, gastric/hepatic events, hypersensitivity events, infections and infestations, and neoplasms.

Anti-drug antibodies (ADAs) and neutralizing antibodies (NAbs) were tested at baseline and weeks 2, 6, 12, and 26. Serum samples were analyzed by using a tiered approach of laboratory screening, confirmation, and titer determination. Serum samples were analyzed for ADA at QPS, LLC (Newark, DE, USA) by using a single validated electrochemiluminescent immunoassay. ADA-positive samples were then tested for neutralizing activity with a validated cell-based assay using PF-06410293 as the capture agent.

PK serum samples were obtained at baseline and weeks 1, 2, 6, 12, and 26 and evaluated for PF-06410293 or adalimumab-EU concentrations by using a validated, sensitive, and specific enzyme-linked immunosorbent assay with a lower limit of quantification of 250 ng/mL (QPS). The prespecified PD marker was hs-CRP.

### Statistical methods

With the assumption of a week-12 ACR20 response rate of 60% for both PF-06410293 and adalimumab-EU, a sample size of 560 patients was determined to provide about 85% power to demonstrate therapeutic equivalence between the treatment arms, and the symmetric margin of ±14% was used for the primary endpoint. This equivalence margin was derived from a meta-analysis of published data from registration studies for adalimumab in patients with RA [2, 3, 17, 18] and was endorsed by both the European Medicines Agency and the Pharmaceuticals and Medical Devices Agency. Exact methods were used to calculate the confidence interval (CI) for the treatment difference in primary efficacy endpoint of week-12 ACR20, using non-responder imputation (NRI) for missing data and for patients with permanent discontinuation of study drug prior to week 12. Therapeutic equivalence was concluded if the two-sided 95% CI for the treatment difference was entirely contained within ±14% margin and additionally if the two-sided 90% CI for the same treatment difference was within the asymmetric margin of −12% to 15% (as requested by the FDA).

The intention-to-treat (ITT) population, defined as all randomly assigned patients, was the primary analysis population. Sensitivity analyses of the primary and secondary endpoints used the per protocol (PP) population, defined as all patients who received study treatment up to week 12, had a week-12 evaluation, and had no major protocol deviations. The DAS28–4(CRP) change from baseline was analyzed by using an analysis of covariance for repeated-measures data approach.

Safety and immunogenicity analyses were performed for the safety population (defined as randomly assigned patients who received any study treatment) on the prespecified TEAEs of special interest and categories of special interest with risk differences (RDs) and 95% CIs by using the asymptotic approach of Miettinen and Nurminen [19]. Transient ADA response after treatment (including the follow-up period) was defined as either a single positive ADA result or two positive sampling time points where the first and last ADA-positive samples (irrespective of any negative samples in between) were separated by less than 16 weeks and the patient’s last ADA sampling time result was negative [20]. PK analysis was conducted for all dosed patients who provided at least one post-dose drug concentration measurement and was summarized by treatment and ADA status by using descriptive statistics (mean, standard deviation [SD], median, and minimum and maximum). PD analysis using hs-CRP concentration over time was summarized by descriptive statistics according to treatment.

## Results

### Patient disposition and demographics

In total, 1231 patients were screened and 597 eligible patients—297 to PF-06410293 and 300 to adalimumab-EU—were randomly assigned to receive study treatment (Additional file 3). Low hs-CRP level was the main reason for screen failure. The safety population included 596 patients, and one adalimumab-EU patient was randomly assigned and not dosed. In both treatment arms, the median duration of study treatment was 24.1 weeks. The first treatment period to week 26 was completed by 286 (96.3%) out of 297 patients in the PF-06410293 arm and 273 (91.0%) out of 300 in the adalimumab-EU arm. Overall, 30 (10.1%) out of 297 patients in the PF-06410293 arm and 46 (15.3%) out of 300 in the adalimumab-EU arm were excluded from the PP population. In most cases, exclusion was due to incomplete study drug dosing up to week 12 for 16 (5.4%) out of 297 patients in the PF-06410293 arm and 34 (11.3%) out of 300 in the adalimumab-EU arm. In the PF-06410293 arm, 29 (9.8%) out of 297 patients, compared with 51 (17.1%) out of 299 in the adalimumab-EU arm, missed one or more doses. This included 18 (6.1%) out of 297 and 34 (11.4%) out of 299 patients who missed one or more doses because of an AE in the PF-06410293 and adalimumab-EU arms, respectively.

Patient demographic and baseline RA characteristics were similar between the treatment arms (Table 1). At baseline, patients had a mean age of 52.5 years, 78.7% were female, and the mean RA duration was 6.8 years. Mean baseline swollen joint counts were 15.4 versus 17.0 and tender joint counts were 24.3 versus 26.7 in the PF-06410293 and adalimumab-EU arms, respectively. Mean baseline DAS28–4(CRP) values were 5.9 (PF-06410293) and 6.1 (adalimumab-EU). Across the two arms, the mean MTX dose was 15.2 mg/week and 55.9% of patients were receiving oral corticosteroids (Table 1).

**Table 1:** Baseline patient demographic and clinical characteristics (ITT population)

	PF-06410293 *n* = 297	Adalimumab-EU *n* = 300
Demographics^a^		
Gender, n (%)		
Female	241 (81.1)	229 (76.3)
Male	56 (18.9)	71 (23.7)
Age, mean (SD), years	51.5 (13.6)	53.5 (12.9)
Weight, mean (SD), kg	74.7 (17.5)	76.2 (20.8)
Body mass index, mean (SD), kg/m^2^	27.5 (6.1)	28.1 (7.3)
Race, n (%)		
White	261 (87.9)	256 (85.3)
Black	6 (2.0)	9 (3.0)
Asian	16 (5.4)	17 (5.7)
Other	14 (4.7)	18 (6.0)
Ethnicity, n (%)		
Hispanic/Latino	25 (8.4)	29 (9.7)
Not Hispanic/Latino	272 (91.6)	271 (90.3)
Clinical characteristics		
RA duration, mean (SD), years	6.8 (7.2)	6.8 (6.9)
Positive RF or anti-CCP antibody or both, n (%)	242 (81.5)	245 (81.7)
Swollen joint count, mean (SD)	15.4 (7.8)	17.0 (9.8)
Tender joint count, mean (SD)	24.3 (12.3)	26.7 (14.8)
hs-CRP, mg/L		
Mean (SD)	21.3 (22.7)	22.8 (25.2)
Median (range)	14.7 (0.2–169)	16.0 (0.2–192)
DAS28–4(CRP), mean (SD)	5.9 (0.9)	6.1 (0.9)
HAQ-DI, mean (SD)	1.5 (0.6)	1.7 (0.6)
Prior use of one biologic drug, n (%)	8 (2.7)	5 (1.7)
Number of prior and current non-biologic DMARDs (in addition to MTX), mean (SD)	1.5 (0.9)	1.5 (0.9)
MTX dose, mean (SD), mg/week	15.2 (4.4)	15.2 (4.5)
Corticosteroid use, n (%)	164 (55.2)	170 (56.7)

*Abbreviations:* *Adalimumab-EU* adalimumab sourced from the European Union, *CCP* cyclic citrullinated peptide, *DAS28–4(CRP)* Disease Activity Score-28: four components based on high-sensitivity C-reactive protein, *DMARD* disease-modifying anti-rheumatic drug, *HAQ-DI* Health Assessment Questionnaire Disability Index, *hs-CRP* high-sensitivity C-reactive protein, *ITT* intention-to-treat, *MTX* methotrexate, *n* number of patients in each category, *RA* rheumatoid arthritis, *RF* rheumatoid factor, *SD* standard deviation

^a^Randomization stratified by geographic region (North America and Western Europe; Japan; Republic of Korea and Taiwan; Latin America; rest of world)

### Efficacy

#### Primary endpoint

**Fig. 2:**
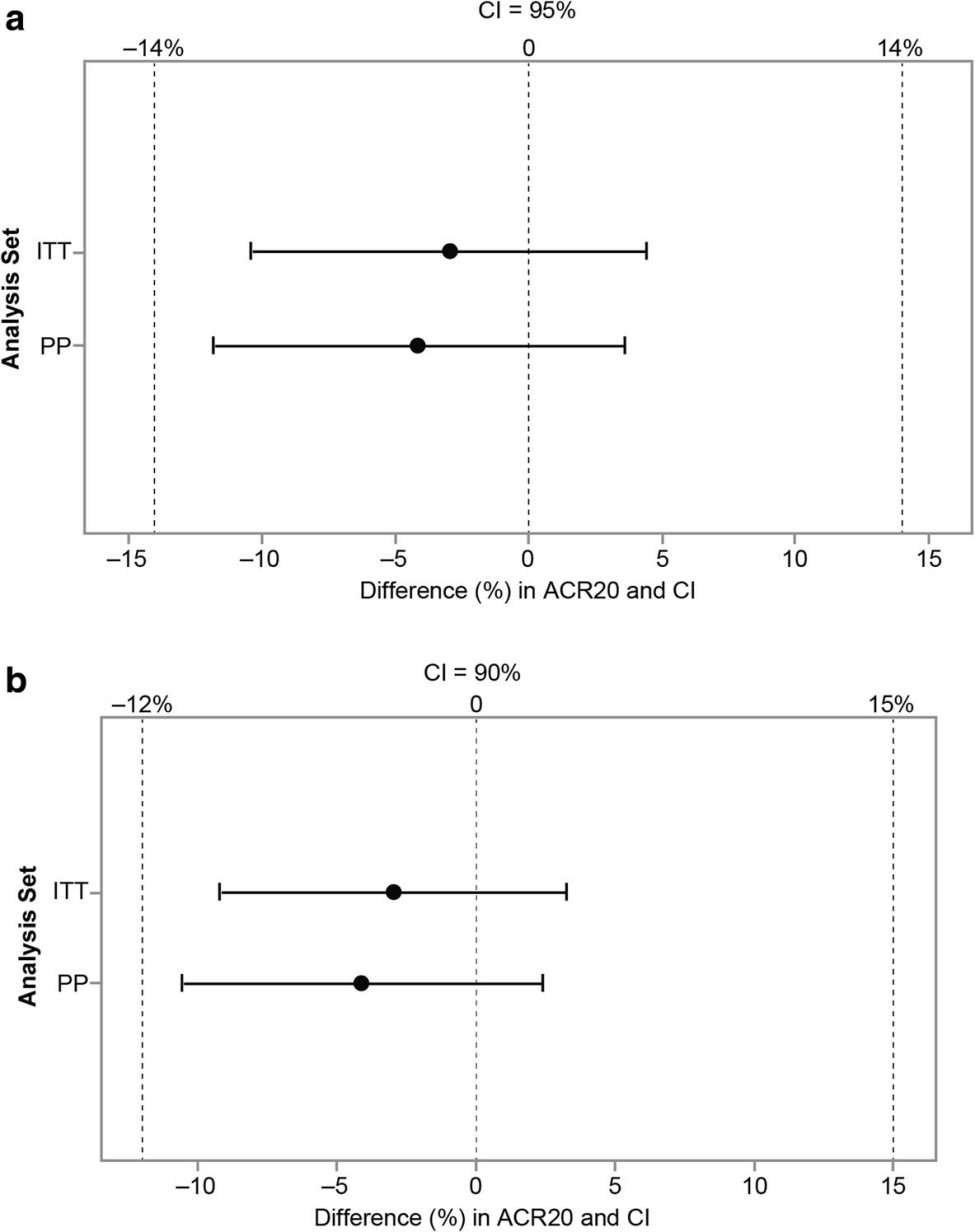
Primary efficacy endpoint of ACR20 at week 12 (with non-responder imputation). **a** Difference (95% CI) between PF-06410293 and adalimumab-EU using a symmetric equivalence margin. **b** Difference (90% CI) between PF-06410293 and adalimumab-EU using an asymmetric equivalence margin. Abbreviations: *ACR20* American College of Rheumatology 20% improvement, *Adalimumab-EU* adalimumab sourced from the European Union, *CI* confidence interval, *ITT* intention-to-treat, *PP* per protocol

Based on the primary efficacy endpoint of ACR20 response rate at week 12, therapeutic equivalence between PF-06410293 and adalimumab-EU was demonstrated by using both prespecified equivalence margins. With observed data in the ITT population, 204 (68.7%) out of 297 patients in the PF-06410293 arm and 218 (72.7%) out of 300 in the adalimumab-EU arm achieved an ACR20 response at week 12, and treatment difference was −3.98%. For the ITT population, response was imputed as non-responder in 19 patients, the treatment difference was −2.98%, based on ACR20 response in 203 (68.4%) out of 297 patients in the PF-06410293 arm and 214 (71.3%) out of 300 in the adalimumab-EU arm, and the 95% CI (−10.38%, 4.44%) was entirely contained within the symmetric margin (Fig. 2a) and 90% CI (−9.25%, 3.28%) was entirely contained within the asymmetric margin (Fig. 2b).

For the PP population sensitivity analysis, 189 (71.1%) out of 266 patients in the PF-06410293 arm and 191 (75.2%) out of 254 in the adalimumab-EU arm achieved an ACR20 response at week 12. The treatment difference was −4.14%, and the corresponding 95% (−11.79%, 3.61%) and 90% (−10.60%, 2.38%) CIs were entirely contained within the symmetric (±14%) and asymmetric (−12%, 15%) equivalence margins, respectively. Other sensitivity analyses of the primary endpoint, including an analysis adjusting for the stratification variable of geographic region and a multiple imputation-based tipping point analysis for missing data, were consistent with the primary result of therapeutic equivalence between PF-06410293 and adalimumab-EU (Additional file 4).

The ACR20 rates at week 12 for subgroups were numerically higher in ADA-negative (70.9% and 77.2%) compared with ADA-positive (63.7% and 65.7%) patients for the PF-06410293 and adalimumab-EU arms, respectively, defined as subjects with a positive ADA test in the first 26 weeks. ACR20 rates for NAb-negative patients were also numerically higher (70.9% and 74.0%) as compared with NAb-positive patients (50.0% and 64.0%) for the PF-06410293 and adalimumab-EU arms, respectively.

#### Secondary endpoints

**Fig. 3:**
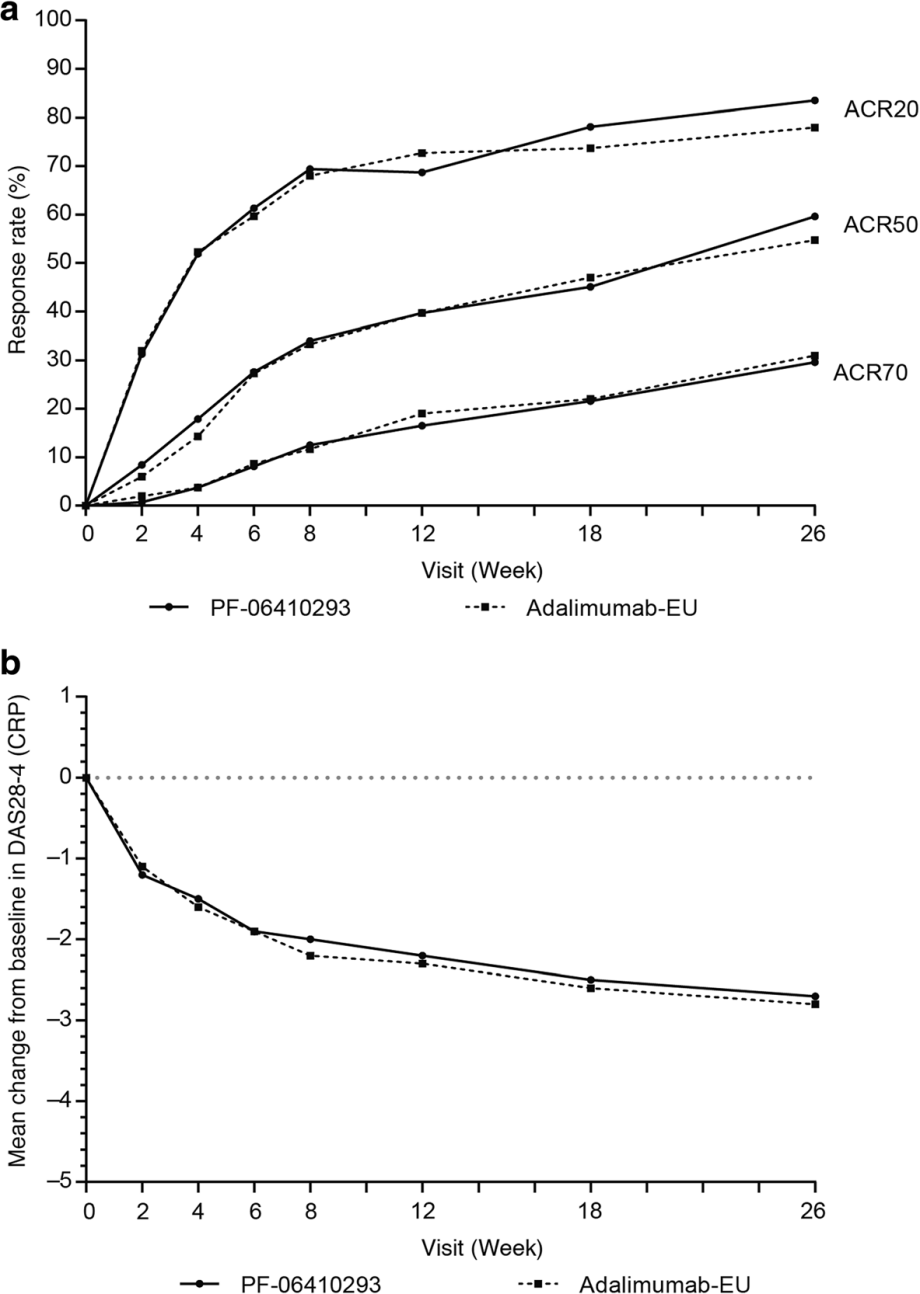
Secondary efficacy endpoints (intention-to-treat population). **a** ACR20/50/70 response rates by study visit. **b** Mean change from baseline in DAS28–4(CRP) by study visit. Abbreviations: *Adalimumab-EU* adalimumab sourced from the European Union, *ACR20/50/70* American College of Rheumatology 20%/50%/70% improvement, *DAS28–4(CRP)* Disease Activity Score-28: four components based on high-sensitivity C-reactive protein

The ACR20/50/70 response rates through week 26 were similar between the PF-06410293 and adalimumab-EU arms (Fig. 3a). Mean changes from baseline in DAS28–4(CRP) were similar between treatment arms at each study visit, and the changes from baseline at week 26 were −2.7 for the PF-06410293 arm and −2.8 for the adalimumab-EU arm (Fig. 3b). At week 26, 162 (54.5%) out of 297 and 147 (49.0%) out of 300 of patients had a good EULAR response in the PF-06410293 and adalimumab-EU arms, respectively (Additional file 5). In the PF-06410293 arm, 87 (29.3%) out of 297 patients achieved DAS28–4(CRP) of less than 2.6 at week 26 compared with 99 (33.0%) out of 300 in the adalimumab-EU arm (Additional file 6). A total of 38 (12.8%) out of 297 patients in the PF-06410293 arm and 44 (14.7%) out of 300 in the adalimumab-EU arm achieved ACR/EULAR remission at week 26, including 26 (8.8%) out of 297 and 27 (9.0%) out of 300 using only the Boolean definition (Additional file 6). At week 26, mean HAQ-DI decreased from baseline by 0.654 in the PF-06410293 arm and by 0.674 in the adalimumab-EU arm (Additional file 7).

### Safety

A total of 143 (48.1%) out of 297 patients in the PF-06410293 arm and 143 (47.8%) out of 299 in the adalimumab-EU arm reported one or more TEAEs. The System Organ Classes (SOCs) with the highest proportion of patients with AEs were infections and infestations in 24.9% and 25.1%, musculoskeletal and connective tissue disorders in 10.4% and 8.7%, and investigations in 8.8% and 7.7% for the PF-06410293 and adalimumab-EU patients, respectively. The number of patients who permanently discontinued treatment because of TEAEs was 11 (3.7%) versus 14 (4.7%) and the number of patients who temporarily discontinued treatment because of TEAEs was 17 (5.7%) versus 29 (9.7%) in the PF-06410293 and adalimumab-EU arms, respectively.

Serious adverse events (SAEs) were reported by 4.0% (PF-06410293) and 4.3% (adalimumab-EU) of patients (Table 2). This included one death due to myocardial infarction in the adalimumab-EU arm. The SOC with the highest proportion of patients with SAEs was infections and infestations, occurring in three patients in each treatment arm.

**Table 2:** All-causality treatment-emergent adverse events (safety population)

	PF-06410293 *n* = 297	Adalimumab-EU *n* = 299
Number of AEs	343	379
Patients with events, n (%)		
AEs	143 (48.1)	143 (47.8)
SAEs	12 (4.0)	13 (4.3)
Grade 3 AEs	15 (5.1)	16 (5.4)^a^
Grade 4 AEs	2 (0.7)	4 (1.3)
Grade 5 AEs	0	1 (0.3)
Patients with temporary treatment discontinuation due to AEs, n (%)	17 (5.7)	29 (9.7)
Patients discontinued from treatment due to AEs^c^, n (%)	11 (3.7)^b^	14 (4.7)
Patients discontinued from the study due to AEs, n (%)	8 (2.7)	9 (3.0)

AEs were graded in accordance with National Cancer Institute Common Terminology Criteria for Adverse Events version 4.03. Grade 1–5 AEs are defined as mild, moderate, severe, life-threatening AEs, and death related to AE, respectively.

*Abbreviations:* *Adalimumab-EU* adalimumab sourced from the European Union, *AE* adverse event, *SAE* serious adverse event

^a^One patient had an AE of neutropenia incorrectly recorded as grade 2; the correct severity was grade 3 (not corrected in this table)

^b^One patient was incorrectly recorded as treatment discontinuation due to an AE; the correct reason was insufficient clinical response (not corrected in this table)

^c^The System Organ Class with the highest proportion of subjects who had AEs leading to permanent treatment discontinuation was infections and infestations (8 [2.7%] subjects on PF-06410293 and 3 [1.0%] subjects on adalimumab-EU)

In total, 5.7% of patients in the PF-06410293 arm and 7.0% in the adalimumab-EU arm reported TEAEs of grade 3 or higher. All-causality grade 4 TEAEs were reported in two patients in the PF-06410293 arm (intentional self-injury, and hemorrhoids with rectal hemorrhage and resulting anemia) and four patients in the adalimumab-EU arm (atrial fibrillation, ileus secondary to colon cancer, gastroenteritis, and papillary thyroid cancer).

The most frequently reported TEAEs occurring in at least 2% of patients in any treatment arm were viral upper respiratory tract infections, increased alanine aminotransferase, hypertension, and headaches (Additional file 8).

Of the TEAEs of special interest, ISRs were reported by five (1.7%) and six (2.0%) patients in the PF-06410293 and adalimumab-EU arms, respectively (Table 3). The primary symptom was redness (three patients in the PF-06410293 arm and two in the adalimumab-EU arm). In addition, one patient in each arm reported pain and swelling. No patients discontinued treatment because of an ISR. For one patient in the PF-06410293 arm and two patients in the adalimumab-EU arm, the ISR occurred on or after the date the patient first tested positive for ADA.

**Table 3:** All-causality treatment-emergent adverse events of special interest with risk difference (safety population)

Event of special interest	PF-06410293 *n* = 297 n (%)	Adalimumab-EU *n* = 299 n (%)	Risk difference (95% CI) (%)
Injection-site reactions	5 (1.7)	6 (2.0)	−0.32 (−2.84, 2.12)
Opportunistic infections	7 (2.4)	5 (1.7)	0.69 (−1.80, 3.32)
Herpes zoster	1 (0.3)	3 (1.0)	−0.67 (−2.61, 0.97)
Latent tuberculosis	5 (1.7)	1 (0.3)	1.35 (−0.35, 3.59)
Confirmed active tuberculosis	0	0	0 (NA)
Oral candidiasis	0	1 (0.3)	−0.33 (−1.87, 0.95)
*Pneumocystis jirovecii* pneumonia	1 (0.3)	0	0.34 (−0.94, 1.88)
Urticaria, angioedema, anaphylactic reaction^a^	0	2 (0.7)	−0.67 (−2.41, 0.61)

*Abbreviations:* *Adalimumab-EU* adalimumab sourced from the European Union, *CI* confidence interval, *NA* not applicable

^a^Only urticaria reported

Overall infection rates were similar at 24.9% and 25.1% for the PF-06410293 and adalimumab-EU arms, respectively. Opportunistic infections (predefined in the study as including latent TB) were reported by seven (2.4%) in the PF-06410293 arm and five (1.7%) patients in the adalimumab-EU arm (Table 3). One case of herpes zoster was reported in the PF-06410293 arm and three cases in the adalimumab-EU arm. Five and one cases of seroconversion with a subsequent diagnosis of latent TB (based on specialist consultation following a positive week-26 QuantiFERON-TB Gold test result) were reported in the PF-06410293 and adalimumab-EU arms, respectively. The RD for latent TB (1.35, 95% CI −0.35, 3.59) was not statistically significant. In total, 5.6% of patients in the PF-06410293 arm and 4.9% in the adalimumab-EU arm had a negative QuantiFERON-TB test at screening and a positive test at week 26. There were no cases of active TB in any patient in either treatment arm. One case of oral candidiasis (adalimumab-EU) and one case of *Pneumocystis jirovecii* pneumonia (PF-06410293) were reported. Overall, pneumonia was reported by 0.7% and 2.0% of the PF-06410293 and adalimumab-EU arms, respectively. There were no cases of anaphylaxis or angioedema in either treatment arm; two cases of urticaria were reported in the adalimumab-EU arm.

A total of 39.1% of patients in each arm reported 183 (PF-06410293) and 202 (adalimumab-EU) AEs in one or more prespecified TEAE categories of interest (Table 4). Hypersensitivity TEAEs were reported in 13 (4.4%) out of 297 patients in the PF-06410293 arm compared with 25 (8.4%) out of 299 in the adalimumab-EU arm (RD −3.98, 95% CI −8.15, −0.06). The most frequently reported hypersensitivity TEAEs were cough (5 versus 3), erythema (4 versus 1), and rash (1 versus 3) in the PF-06410293 and adalimumab-EU arms, respectively. Hypersensitivity TEAEs occurring on or after the date a patient first tested positive for ADA included six AEs reported by five patients in the PF-06410293 arm and nine AEs reported by seven patients in the adalimumab-EU arm. Two patients in the PF-06410293 arm reported grade 3 hypersensitivity SAEs, including interstitial lung disease and toxic skin eruption. The rate of blood and lymphatic system events was numerically higher in the PF-06410293 versus adalimumab-EU arms—22 (7.4%) out of 297 versus 14 (4.7%) out of 299—but this was not statistically significant (RD 2.73, 95% CI −1.15, 6.79). The reported malignancies included one (basal cell carcinoma) and two (adenocarcinoma of the colon and papillary thyroid cancer) patients in the PF-06410293 and adalimumab-EU arms, respectively.

**Table 4:** Prespecified treatment-emergent adverse event categories of interest with risk difference (safety population)

Category	PF-06410293 *n* = 297 n (%)	Adalimumab-EU *n* = 299 n (%)	Risk difference (95% CI) (%)
Blood and lymphatic system events	22 (7.4)	14 (4.7)	2.73 (−1.15, 6.79)
Cardiovascular events	9 (3.0)	16 (5.4)	−2.32 (−5.84, 0.96)
Demyelinating conditions	0	0	0 (NA)
Gastric/hepatic events	11 (3.7)	14 (4.7)	−0.98 (−4.44, 2.39)
Hypersensitivity^a^	13 (4.4)	25 (8.4)	−3.98 (−8.15, −0.06)
Infections and infestations	74 (24.9)	75 (25.1)	−0.17 (−7.13, 6.80)
Neoplasms	5 (1.7)	5 (1.7)	0.01 (−2.38, 2.42)
Other^b^	11 (3.7)	10 (3.3)	0.36 (−2.80, 3.57)

*Abbreviations:* *Adalimumab-EU* adalimumab sourced from the European Union, *CI* confidence interval, *MedDRA* Medical Dictionary for Regulatory Activities, *NA* not applicable

^a^Hypersensitivity events identified by Hypersensitivity Standardized MedDRA Query (broad and narrow), Anaphylactic reactions Standardized MedDRA Query (broad and narrow), and High-Level Group Terms Immunology and allergy investigations

^b^Other events identified by High-Level Group Terms Skin Vascular Abnormalities, Central Nervous System Vascular Abnormalities and Medication Errors; Higher Level Terms Connective Tissue Disorders, Vasculitides (not elsewhere classified), Rashes, Eruptions and Exanthems (not elsewhere classified); Lower Level Teams Seizure and Convulsions; and Preferred Terms Lupus-like Syndrome, Headache and Migraine

### Immunogenicity, PK, and PD

**Fig. 4:**
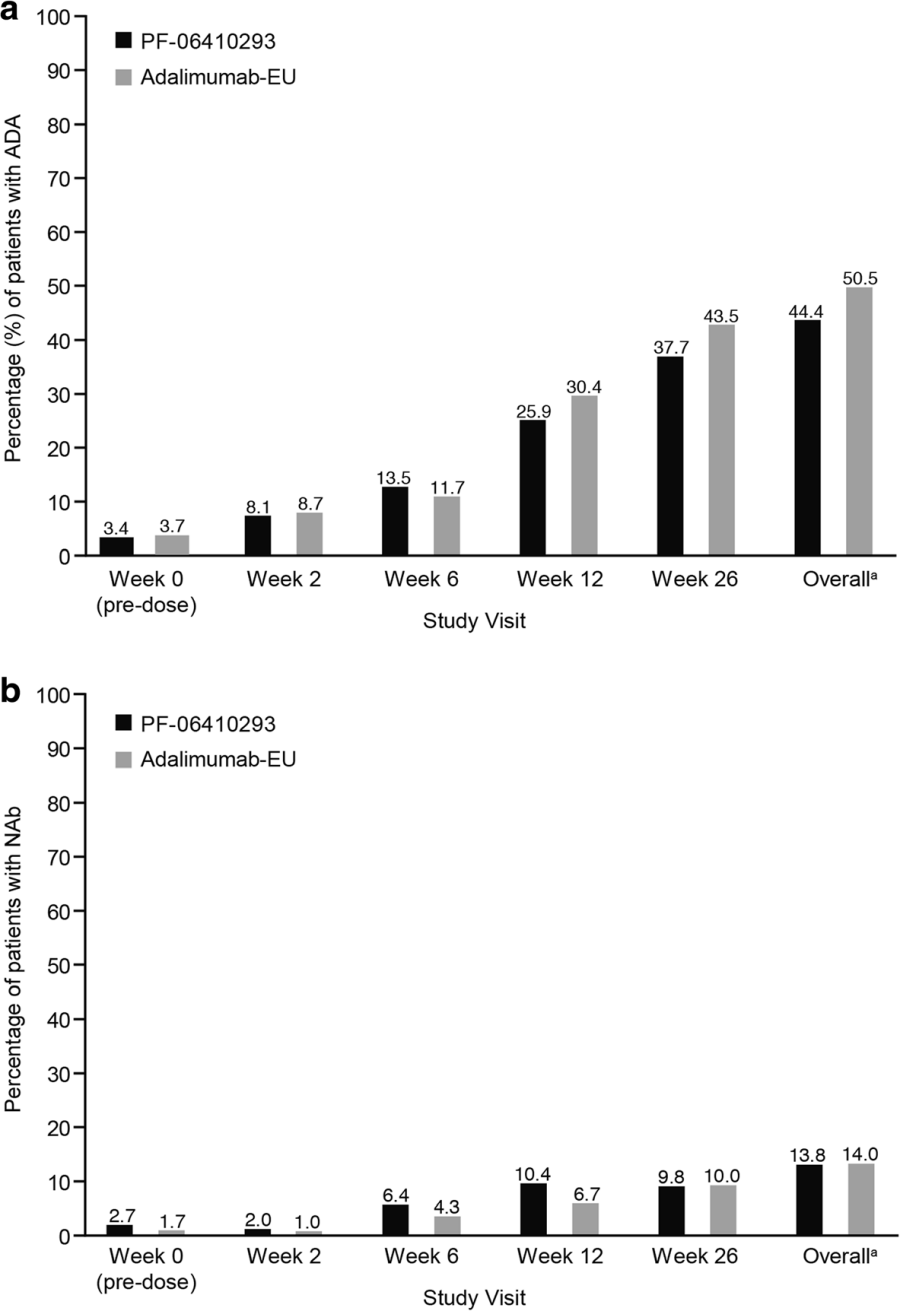
ADA and NAb incidence by study visit (safety population). **a** ADA incidence. **b** NAb incidence. The percentage of NAb-positive patients is based on the total number of patients in each treatment group. ^a^“Overall” includes data from week 2, week 6, week 12, week 26, end-of-treatment/early termination, follow-up, and unplanned visits in treatment period 1. Abbreviations: *ADA* anti-drug antibody, *Adalimumab-EU* adalimumab sourced from the European Union, *NAb* neutralizing antibody

Overall, 44.4% and 50.5% of patients in the PF-06410293 and adalimumab-EU treatment arms, respectively, had at least one post-dose sample that tested positive for ADA (Fig. 4; Additional file 9). ADAs were transient in 11.4% of patients in the PF-06410293 arm and in 6.0% in the adalimumab-EU arm. Of the ADA-positive patients, 31.1% in the PF-06410293 and 27.8% in the adalimumab-EU treatment arms tested positive for NAb.

The mean serum drug trough concentrations at week 26 were 8244 and 7190 ng/mL in the PF-06410293 and adalimumab-EU arms, respectively. Mean serum concentrations of both drugs were lower in ADA-positive patients (4683 and 4041 ng/mL) compared with ADA-negative patients (11,090 and 10,460 ng/mL) for the PF-06410293 and adalimumab-EU arms, respectively. The mean hs-CRP concentrations (prespecified PD marker) were decreased at week 26 in both arms (ITT population); the changes from baseline to week 26 were −11.1 (PF-06410293) and −13.6 mg/L (adalimumab-EU).

## Discussion

This comparative clinical study was conducted to evaluate the biosimilarity of PF-06410293 and adalimumab-EU. The primary goal of the study was met by demonstrating therapeutic equivalence of PF-06410293 and adalimumab-EU using the symmetric and asymmetric margins for the week-12 ACR20 primary endpoint comparison. Sensitivity analyses of the primary endpoint supported a conclusion of therapeutic equivalence. As expected, ACR20 response rates at week 12 were numerically higher in ADA-negative patients compared with ADA-positive in both treatment arms. Secondary endpoints reported up to week 26, including ACR50, ACR70, change from baseline in DAS28–4(CRP), EULAR response, DAS28–4(CRP) of less than 2.6, ACR/EULAR remission, and HAQ-DI all supported therapeutic equivalence.

The safety profiles of PF-06410293 and adalimumab-EU to week 26 were comparable, including similar findings with respect to the number of AEs, SAEs, and prespecified TEAEs and TEAE categories of special interest. The only statistically significant safety difference was a lower rate of hypersensitivity events observed in the PF-06410293 arm compared with the adalimumab-EU arm; however, no correction for multiplicity was performed in this study. As might be expected in a single clinical trial, numerical differences were observed between the treatment arms, including an imbalance in the development of latent TB (more common in the PF-06410293 group) and hypersensitivity events (more common in the adalimumab-EU group). Of note, the diagnosis of latent TB was based on investigator judgment, local practice, and consultation with a pulmonary or infectious disease specialist and after a protocol-mandated QuantiFERON-TB test was performed. The percentage of patients who converted to a QuantiFERON-TB test positive at week 26 was balanced between the treatment arms, suggesting that there was no clinically meaningful difference in the rate of QuantiFERON-TB test conversion between PF-06410293 and adalimumab-EU. The safety profile for both study drugs appears to be consistent with the known safety profile of reference adalimumab-EU.

The immunogenicity profiles observed during the first 26 weeks of treatment were similar for the two treatment arms, and there was a somewhat lower incidence of patients testing positive for ADA in the PF-06410293 arm. Serum drug concentrations were numerically higher in the PF-06410293 arm; however, these differences were not considered clinically meaningful, as the clinical response was similar in the two treatment arms. The hs-CRP response as a PD biomarker supports this lack of clinical significance, as the decrease in hs-CRP was similar for the two arms over the first 26 weeks of treatment. As expected, the serum drug concentrations of PF-06410293 and adalimumab-EU were lower in both treatment arms for ADA-positive compared with ADA-negative patients.

## Conclusions

Results from the first 26 weeks of dosing demonstrated no clinically meaningful differences in efficacy, safety, immunogenicity, PK, or PD between PF-06410293 and adalimumab-EU in patients with active RA. Upcoming data from the subsequent 6 months of the trial will provide additional efficacy, safety, and immunogenicity information, including data on patients after a blinded transition from adalimumab-EU to PF-06410293 and those who receive a total of 1 year of treatment with either PF-06410293 or adalimumab-EU.

## Additional files

Additional file 1: Inclusion criteria for study enrollment. (DOCX 48 kb)

Additional file 2: Exclusion criteria. (DOCX 53 kb)

Additional file 3: Consolidated Standards of Reporting Trials (CONSORT) flow diagram of patient progress in the two treatment arms. ^a^Patients may screen fail for more than one reason. ^b^Patients may be excluded from the per-protocol population for more than one reason. Abbreviation: *Adalimumab-EU* adalimumab sourced from the European Union. (PNG 171 kb)

Additional file 4: Multiple imputation-based tipping-point analysis. (DOCX 48 kb)

Additional file 5: EULAR response by study visit (ITT population). Abbreviations: *EULAR* European League Against Rheumatism, *ITT* intention-to-treat. (DOCX 48 kb)

Additional file 6: DAS28–4(CRP) of less than 2.6 and ACR/EULAR remission by study visit (ITT population). Abbreviations: *ACR/EULAR* American College of Rheumatology/European League Against Rheumatism, *DAS28–4(CRP)* Disease Activity Score 28 joints: four components based on high-sensitivity C-reactive protein, *ITT* intention-to-treat. (DOCX 50 kb)

Additional file 7: Mean change from baseline in HAQ-DI by visit (ITT population). Abbreviations: *Adalimumab-EU* adalimumab sourced from the European Union, *HAQ-DI* health assessment questionnaire disability index, *ITT* intention-to-treat (PNG 84 kb)

Additional file 8: All-causality treatment-emergent adverse events in at least 2% of patients in any treatment arm (safety population). (DOCX 49 kb)

Additional file 9: ADA and NAb incidence by study visit (safety population). Abbreviations: *ADA* anti-drug antibody, *NAb* neutralizing antibody. (DOCX 52 kb)

## Abbreviations

ACR: American College of Rheumatology
ACR20: American College of Rheumatology 20% improvement
ADA: Anti-drug antibody
Adalimumab-EU: Adalimumab sourced from the European Union
AE: Adverse event
bDMARD: Biologic disease-modifying anti-rheumatic drug
CI: Confidence interval
DAS28–4(CRP): Disease Activity Score 28 joints: four components based on high-sensitivity C-reactive protein
DMARD: Disease-modifying anti-rheumatic drug
ESR: Erythrocyte sedimentation rate
EULAR: European League Against Rheumatism
FDA: US Food and Drug Administration
HAQ-DI: Health Assessment Questionnaire Disability Index
hs-CRP: High-sensitivity C-reactive protein
ISR: Injection-site reaction
ITT: Intention-to-treat
MTX: Methotrexate
NAb: Neutralizing antibody
PD: Pharmacodynamics
PK: Pharmacokinetics
PP: Per protocol
RA: Rheumatoid arthritis
RD: Risk difference
SAE: Serious adverse event
SOC: System Organ Class
TB: Tuberculosis
TEAE: Treatment-emergent adverse event
TNF: Tumor necrosis factor
