# Supplementary material for: A comparative clinical study of PF-06410293, a candidate adalimumab biosimilar, and adalimumab reference product (Humira®) in the treatment of active rheumatoid arthritis
Source: Arthritis Res Ther. 2018 Aug 15;20:178. doi: 10.1186/s13075-018-1676-y (PMC6094896; doi:10.1186/s13075-018-1676-y)
Supplement: Supplementary file 1 — Inclusion criteria for study enrollment. (DOCX 48 kb) [file 13075_2018_1676_MOESM1_ESM.docx]

# Additional file 1 Inclusion criteria for study enrollment

| 1. | Evidence of a personally signed and dated informed consent document indicating that the patient (or a legally acceptable representative) had been informed of all pertinent aspects of the study. |
| --- | --- |
| 2. | Patients who were willing and able to comply with scheduled visits, treatment plan, laboratory tests, lifestyle guidelines, and other study procedures. |
| 3. | Male and female patients aged ≥18 years at the time of informed consent. Where required by regulations, consent from a legally acceptable representative was required for all patients who were <20 years of age. |
| 4. | Male and female patients of childbearing potential and at risk for pregnancy had to agree to use a highly effective method of contraception throughout the study and for ≥6 months after the last dose of assigned treatment.  Female patients were considered not of childbearing potential if they met ≥1 of the following criteria:   1. Achieved postmenopausal status, defined as follows: cessation of regular menses for ≥12 consecutive months with no alternative pathological or physiological cause; and had a serum follicle-stimulating hormone level within the laboratory’s reference range for postmenopausal women; or 2. Had undergone a documented hysterectomy and/or bilateral oophorectomy; or 3. Had medically confirmed ovarian failure. |
| 5. | Diagnosis of RA based on 2010 ACR/EULAR classification criteria for RA for ≥4 months. |
| 6. | Met Class I, II or III of the ACR 1991 Revised Criteria for Global Functional Status in RA. |
| 7. | Moderately to severely active RA disease as defined by the following criteria:   1. ≥6 tender joints (of 68 assessed) at both screening and baseline, and 2. ≥6 swollen joints (of 66 assessed) at both screening and baseline, and 3. hs-CRP ≥8 mg/L (0.8 mg/dL) at screening, performed by the central laboratory. Patients who did not meet this entry criterion but satisfied all other study entry criteria might have serum hs-CRP concentration re-tested once within 14 days and, if the repeat hs-CRP concentration was ≥8 mg/L (0.8 mg/dL), were eligible to enroll into the study provided all other inclusion/exclusion criteria were met. |
| 8. | Patients had to have received oral, subcutaneous, or intramuscular methotrexate for ≥12 weeks and been on a stable dose for ≥4 weeks prior to first dose of study drug. The stable dose had to be 10–25 mg/week, with the exception of 6–25 mg/week where 6 mg/week was a recommended initial dose by local guidance or standard of care. |
| 9. | Stable dose of oral folic acid (≥1 mg/day on ≥5 days/week) or oral folinic acid (≥5 mg once weekly) supplementation for ≥21 days prior to the first dose of study drug. In countries that did not have approved folic acid 1 mg or folinic acid 5 mg presentations, a regimen of folic acid of ≥5 mg weekly was acceptable. |
| 10. | No current or prior treatment with adalimumab or lymphocyte depleting therapies (e.g. rituximab, Campath [alemtuzumab]). Patients might have received up to 2 doses of 1 biologic therapy (any type), including an anti-TNF inhibitor biologic agent (other than adalimumab), enrolling after a washout period of ≥12 weeks or 5 half-lives prior to the first dose of study drug, whichever was longer. |
| 11. | If receiving an oral corticosteroid, patient had to be on a stable dose of ≤10 mg/day of prednisone (or equivalent) for ≥4 weeks prior to the first dose of study drug. Patient had to not receive any intramuscular or intra-articular corticosteroids within the 4 weeks prior to the first dose of study drug. |
| 12. | If receiving an oral or topical NSAID/COX-2 inhibitor, patient had to be on a stable dose of only 1 NSAID/COX-2 inhibitor drug for ≥4 weeks prior to the first dose of study drug at a dosage less than or equal to the maximum recommended dose in the product information. In addition, a cardiovascular dose of aspirin (≤325 mg/day) was permitted. |

Patients had to meet all of these inclusion criteria to be eligible for enrollment into the study

*ACR* American College of Rheumatology, *COX-2* cyclooxygenase-2, *EULAR* European League Against Rheumatism, *hs-CRP* high-sensitivity C-reactive protein, *NSAID* non-steroidal anti-inflammatory drug, *RA* rheumatoid arthritis, *TNF* tumor necrosis factor
