# Supplementary material for: A comparative clinical study of PF-06410293, a candidate adalimumab biosimilar, and adalimumab reference product (Humira®) in the treatment of active rheumatoid arthritis
Source: Arthritis Res Ther. 2018 Aug 15;20:178. doi: 10.1186/s13075-018-1676-y (PMC6094896; doi:10.1186/s13075-018-1676-y)
Supplement: Supplementary file 2 — Exclusion criteria. (DOCX 53 kb) [file 13075_2018_1676_MOESM2_ESM.docx]

**Additional file 2** Exclusion criteria

| 1. | Pregnant females and breastfeeding females, male patients with partners currently pregnant who were unwilling to use a condom, or male and female patients of childbearing potential who were unwilling or unable to use a highly effective method of contraception as outlined in the protocol for the duration of the study and for ≥6 months after the last dose of study drug. |
| --- | --- |
| 2. | Clinically significant laboratory abnormalities at screening, including but not limited to inadequate bone marrow, liver, renal and immune system function as defined by the following laboratory criteria:   1. Hemoglobin <9 g/dL 2. Absolute neutrophil count ≤1500 cells/mm^3^ 3. White blood cell count <3.0 × 10^9^/L 4. Platelets <100 × 10^9^/L 5. Aspartate aminotransferase/alanine aminotransferase ≥2 × the upper limit of normal 6. Bilirubin ≥1.5 × upper limit of normal 7. Serum creatinine ≥1.5 mg/dL 8. Beta-D-glucan = positive (Japan only)   Patients who did not meet any laboratory entry criteria but satisfied all other study entry criteria might have the laboratory re-tested within 14 days and, if within the required range, were eligible to enroll into the study provided all other inclusion/exclusion criteria were met. |
| 3. | Evidence or history of nervous system demyelinating diseases (including multiple sclerosis, optic neuritis, Guillain-Barré syndrome). History of seizure disorder requiring treatment in the previous 5 years prior to screening. |
| 4. | History of infection as evidenced by:   1. History of recurrent (>1 episode) limited herpes simplex that required current chronic antiviral therapy, or disseminated (a single episode) herpes simplex 2. History of disseminated or recurrent infection with EBV, human papilloma virus, or varicella zoster. A single, limited episode in the past was not exclusionary 3. Infection requiring hospitalization or parenteral antimicrobial therapy judged clinically significant by the investigator within 6 months prior to first dose of study drug 4. History of an infected joint prosthesis at any time. |
| 5. | Known or screen test positive for human immunodeficiency virus, hepatitis B virus, or hepatitis C virus. Patients with an isolated positive HBsAb test with previous hepatitis B vaccination, or both positive HBsAb and HBcAb tests and negative HBsAg, were eligible. |
| 6. | Evidence of untreated or inadequately treated latent or active infection with TB as defined below:   1. Positive TB test at screening, unless prior treatment for latent or active TB (QuantiFERON-TB Gold In-Tube Test). Patients with a positive screening QuantiFERON-TB Gold In-Tube Test required medical monitor approval prior to randomization 2. Patients previously treated for active TB infection, or detected with active TB at screening, had to have completed a successful course of treatment in accordance with local guidelines. Patients currently receiving TB therapy for active TB were excluded 3. Patients previously treated for latent TB infection could be enrolled. Patients detected with latent TB at screening could be enrolled after a minimum of 4 weeks of TB therapy in accordance with local guidelines. |
| 7. | Chest radiography with evidence of active TB, fungal infections, or other clinically significant abnormalities taken at screening or within 12 weeks prior to screening. If the TB had been treated as specified in Exclusion Criteria 6b, a chest radiography result consistent with prior TB would not exclude the patient. |
| 8. | Evidence of current or recent history of uncontrolled, clinically significant hematological, renal, endocrine, pulmonary, gastrointestinal, hepatic, infectious, psychiatric, neurologic, allergic, or cardiovascular disease including evidence or history of moderate or severe heart failure (New York Heart Association Class III/IV) or screening 12-lead electrocardiogram that demonstrated clinically relevant abnormalities which could affect patient safety, and patients who were contraindicated for treatment with adalimumab in accordance with the approved local label. |
| 9. | Evidence or history of a malignancy within the past 5 years (with the exception of adequately treated or excised non-metastatic basal cell or squamous cell cancer of the skin, or cervical carcinoma in situ with no evidence of recurrence), or history of any lymphoproliferative disorder (e.g., EBV-related lymphoproliferative disorder, lymphoma, or leukemia). |
| 10. | History of recurrent inflammatory joint disease other than RA (e.g., post-infectious arthritis, gout, etc.) or history of any other autoimmune rheumatic diseases (e.g., vasculopathies, spondyloarthropathies, etc.) other than Sjögren’s syndrome. |
| 11. | Any current or prior treatment with the following disease-modifying anti-rheumatic drugs within the relevant washout period:   1. Washout for 12 weeks prior to first dose of study drug with immunosuppressive drugs, including alkylating agents (e.g., cyclophosphamide and chlorambucil), mycophenolate mofetil, leflunomide, azathioprine, or 6-mercaptopurine 2. Washout for 8 weeks prior to first dose of study drug with gold therapy including auranofin or injectable gold (aurothioglucose or aurothiomalate), tofacitinib, tacrolimus, cyclosporine, and D-penicillamine 3. Washout for 4 weeks prior to first dose of study drug for sulfasalazine or anti-malarial drugs 4. Washout for at least 4 weeks or 5 half-lives, whichever was longer, prior to first dose of study drug for investigational treatments for RA not specifically mentioned. |
| 12. | Known requirement for treatment with prohibited concomitant medications during the study. |
| 13. | Significant trauma or surgical procedure within 4 weeks prior to first dose of study drug. |
| 14. | Positive urine drug test at screening for substances of abuse that was not due to prescribed medication, or past or current history of addiction to or dependence on non-prescribed substances within 12 months prior to screening. |
| 15. | History of severe allergic or hypersensitivity or anaphylactic reaction to a biologic drug or to active or inactive components of the study drug. |
| 16. | Exposure to any live vaccines within 4 weeks prior to administration of the first dose of study drug, or lack of willingness to avoid exposure to any live vaccines during the trial and for ≥3 months after the last dose of study drug. |
| 17. | Participation in other studies involving investigational drug(s) (Phases 1–4) within ≥4 weeks or 5 half-lives of investigational product, whichever was longer, before the first dose of study drug and/or during the study participation. |
| 18. | Other severe acute or chronic medical or psychiatric condition or laboratory abnormality that might increase the risk associated with study participation or investigational product administration or might interfere with the interpretation of study results and, in the judgment of the investigator, would make the patient inappropriate for entry into this study. |
| 19. | Patients who were investigational site staff members directly involved in the conduct of the study and their family members, site staff members otherwise supervised by the investigator, or patients who were Sponsor employees directly involved in the conduct of the study. |

Patients presenting with any of these exclusion criteria were not included in the study.

*EBV* Epstein Barr virus, *HBcAb* hepatitis B core antibody, *HBsAb* antibody to hepatitis B surface antigen, *HBsAg* hepatitis B surface antigen, *RA* rheumatoid arthritis, *TB* tuberculosis.
