# Supplementary figures and images for: A comparative clinical study of PF-06410293, a candidate adalimumab biosimilar, and adalimumab reference product (Humira®) in the treatment of active rheumatoid arthritis
Source: Arthritis Res Ther. 2018 Aug 15;20:178. doi: 10.1186/s13075-018-1676-y (PMC6094896; doi:10.1186/s13075-018-1676-y)

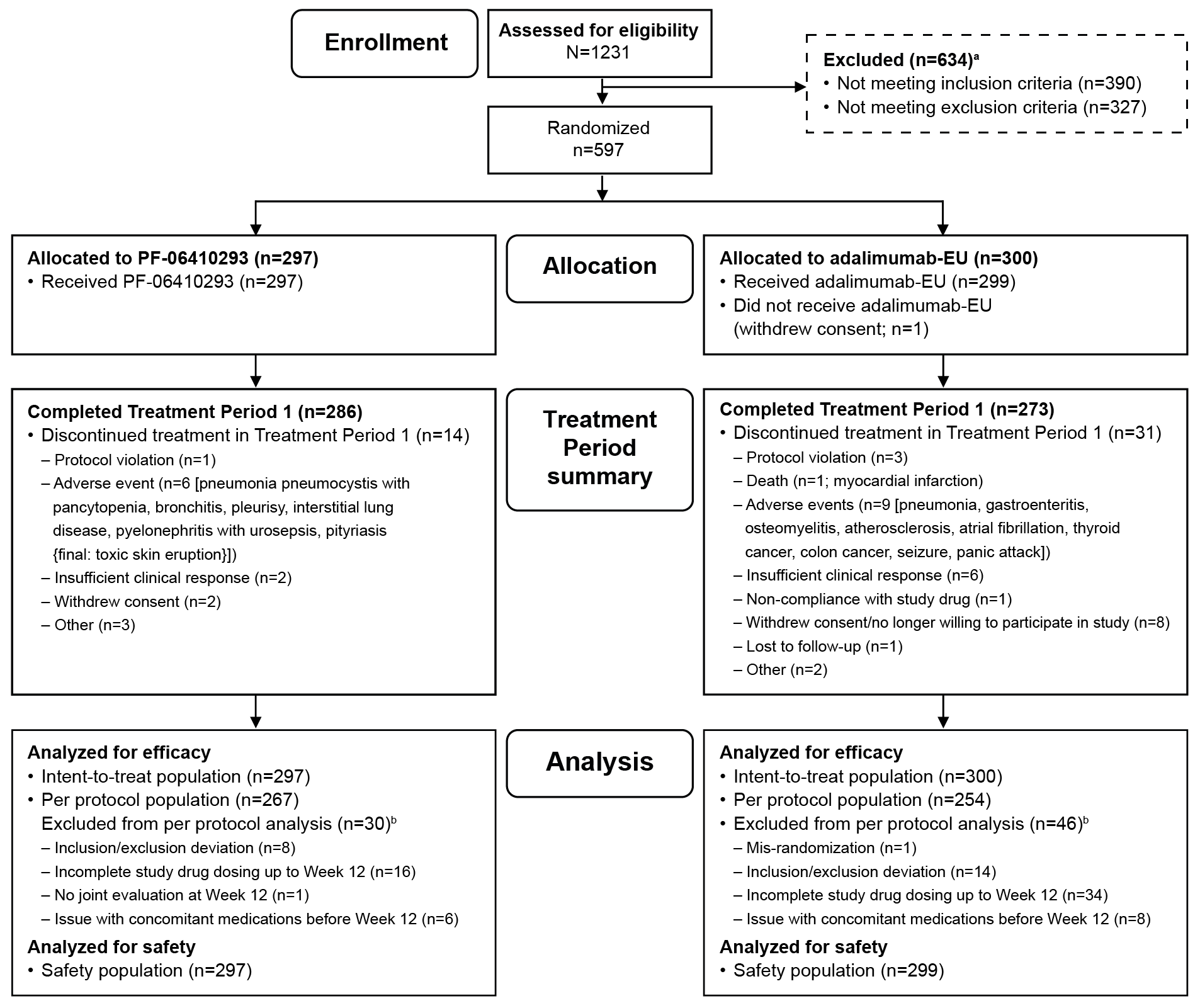

Supplement: Supplementary file 3 — Consolidated Standards of Reporting Trials (CONSORT) flow diagram of patient progress in the two treatment arms. aPatients may screen fail for more than one reason. bPatients may be excluded from the per-protocol population for more than one reason. Abbreviation: Adalimumab-EU adalimumab sourced from the European Union. (PNG 171 kb) [file 13075_2018_1676_MOESM3_ESM.png]

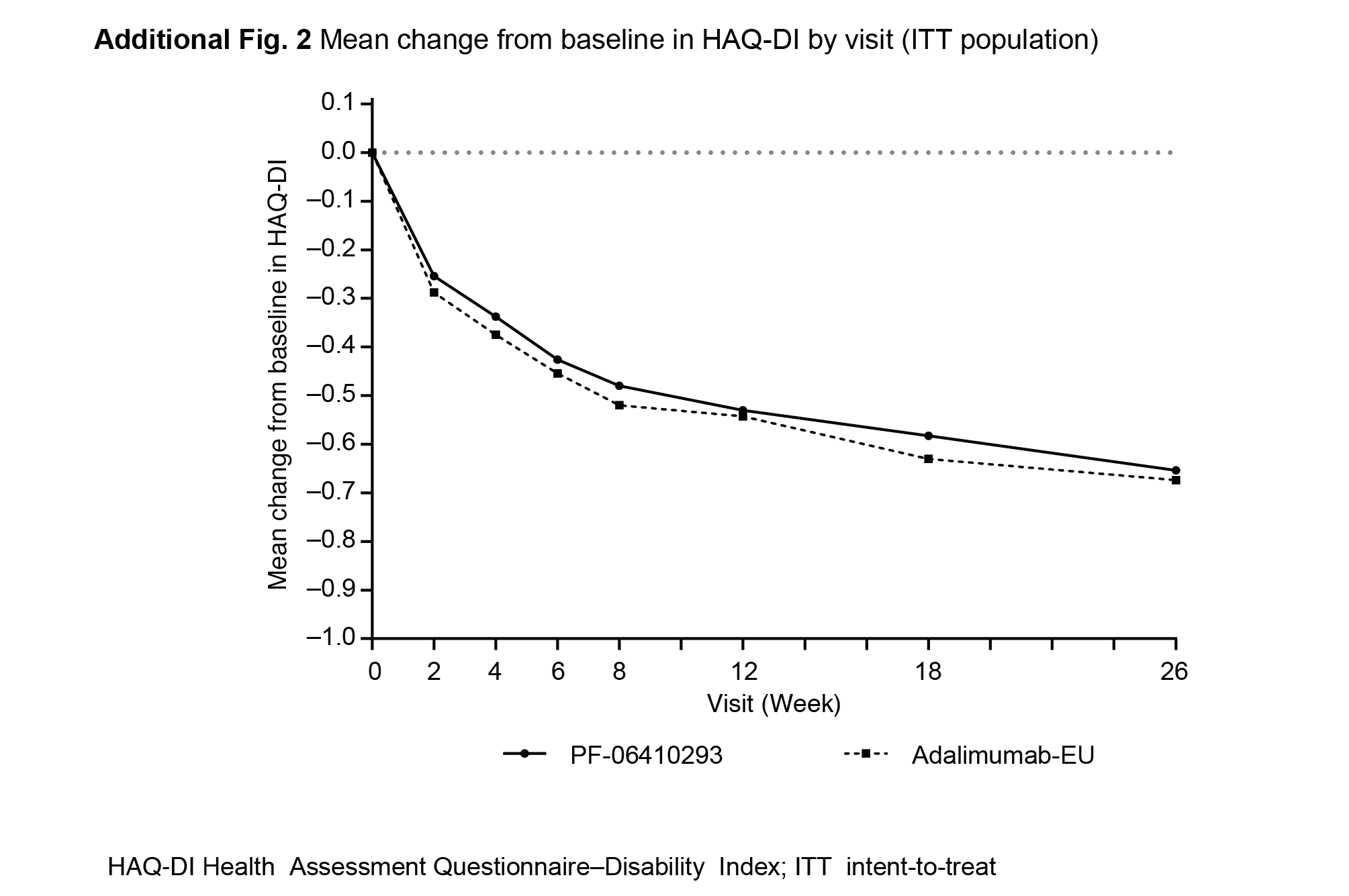

Supplement: Supplementary file 7 — Mean change from baseline in HAQ-DI by visit (ITT population). Abbreviations: Adalimumab-EU adalimumab sourced from the European Union, HAQ-DI health assessment questionnaire disability index, ITT intention-to-treat (PNG 84 kb) [file 13075_2018_1676_MOESM7_ESM.png]
