# Supplementary material for: A comparative clinical study of PF-06410293, a candidate adalimumab biosimilar, and adalimumab reference product (Humira®) in the treatment of active rheumatoid arthritis
Source: Arthritis Res Ther. 2018 Aug 15;20:178. doi: 10.1186/s13075-018-1676-y (PMC6094896; doi:10.1186/s13075-018-1676-y)
Supplement: Supplementary file 4 — Multiple imputation-based tipping-point analysis. (DOCX 48 kb) [file 13075_2018_1676_MOESM4_ESM.docx]

**Additional file 4** Multiple imputation-based tipping-point analysis

Treatment comparison analyses for ACR20 response at Week 12 were performed by assuming all the missing observations in PF-06410293 arm as non-responders and all the missing observations in adalimumab-EU arm as responders, and vice versa. These analyses served as indicators for the need to perform a tipping point analysis for ACR20 response at Week 12. The 95% CIs for the treatment differences were contained within the symmetric margin; therefore, a tipping point analysis was not necessary as the results always remained positive under the symmetric margin.

A tipping-point sensitivity analysis for ACR20 at Week 12 in the ITT population was performed using the asymmetric margin. A series of not-missing-at-random delta adjustments (log odds scale) were applied to the predictive probabilities using multiple imputations to explore departures from missing-at-random (MAR) imputation (delta=0). The exponential-transformed delta [exp (delta)] was applied as a multiplier to the odds ratio governing the treatment difference among patients with missing data at Week 12. The results remained positive (90% CI for the RD in [−12%, 15%]) for all delta values except for extreme departures from MAR [exp (delta) ≤1/500], indicating the robustness of the primary and secondary analyses of ACR20 at Week 12.
