# Supplementary material for: A comparative clinical study of PF-06410293, a candidate adalimumab biosimilar, and adalimumab reference product (Humira®) in the treatment of active rheumatoid arthritis
Source: Arthritis Res Ther. 2018 Aug 15;20:178. doi: 10.1186/s13075-018-1676-y (PMC6094896; doi:10.1186/s13075-018-1676-y)
Supplement: Supplementary file 5 — EULAR response by study visit (ITT population). Abbreviations: EULAR European League Against Rheumatism, ITT intention-to-treat. (DOCX 48 kb) [file 13075_2018_1676_MOESM5_ESM.docx]

**Additional file 5** EULAR response by study visit (ITT population)

| **Study visit** | **EULAR response** | **PF-06410293**  n=297  n (%) | **Adalimumab-EU**  n=300  n (%) |
| --- | --- | --- | --- |
| Week 2 | Good | 18 (6.1) | 20 (6.7) |
|  | Moderate | 161 (54.2) | 136 (45.3) |
|  | None | 110 (37.0) | 133 (44.3) |
| Week 4 | Good | 48 (16.2) | 42 (14.0) |
|  | Moderate | 173 (58.3) | 168 (56.0) |
|  | None | 71 (23.9) | 81 (27.0) |
| Week 6 | Good | 69 (23.2) | 65 (21.7) |
|  | Moderate | 178 (59.9) | 173 (57.7) |
|  | None | 48 (16.2) | 55 (18.3) |
| Week 8 | Good | 93 (31.3) | 89 (29.7) |
|  | Moderate | 160 (53.9) | 160 (53.3) |
|  | None | 38 (12.8) | 43 (14.3) |
| Week 12 | Good | 104 (35.0) | 107 (35.7) |
|  | Moderate | 149 (50.2) | 149 (49.7) |
|  | None | 37 (12.5) | 37 (12.3) |
| Week 18 | Good | 137 (46.1) | 132 (44.0) |
|  | Moderate | 134 (45.1) | 125 (41.7) |
|  | None | 21 (7.1) | 29 (9.7) |
| Week 26 | Good | 162 (54.5) | 147 (49.0) |
|  | Moderate | 110 (37.0) | 102 (34.0) |
|  | None | 16 (5.4) | 27 (9.0) |

*Adalimumab-EU* adalimumab sourced from the European Union, *EULAR* European League Against Rheumatism, *ITT* intent-to-treat
