# Supplementary material for: A comparative clinical study of PF-06410293, a candidate adalimumab biosimilar, and adalimumab reference product (Humira®) in the treatment of active rheumatoid arthritis
Source: Arthritis Res Ther. 2018 Aug 15;20:178. doi: 10.1186/s13075-018-1676-y (PMC6094896; doi:10.1186/s13075-018-1676-y)
Supplement: Supplementary file 6 — DAS28–4(CRP) of less than 2.6 and ACR/EULAR remission by study visit (ITT population). Abbreviations: ACR/EULAR American College of Rheumatology/European League Against Rheumatism, DAS28–4(CRP) Disease Activity Score 28 joints: four components based on high-sensitivity C-reactive protein, ITT intention-to-treat. (DOCX 50 kb) [file 13075_2018_1676_MOESM6_ESM.docx]

**Additional file 6** DAS28-4(CRP) <2.6 and ACR/EULAR remission by study visit (ITT population)

|  |  | Week 2  n (%) | Week 4  n (%) | Week 6  n (%) | Week 8  n (%) | Week 12  n (%) | Week 18  n (%) | Week 26  n (%) |
| --- | --- | --- | --- | --- | --- | --- | --- | --- |
| **PF-06410293**  n=297 | **DAS28-4(CRP) <2.6**  Yes | 9 (3.0) | 20 (6.7) | 35 (11.8) | 49 (16.5) | 63 (21.2) | 71 (23.9) | 87 (29.3) |
|  | **ACR/EULAR remission** |  |  |  |  |  |  |  |
|  | Yes^a^ | 2 (0.7) | 3 (1.0) | 14 (4.7) | 16 (5.4) | 24 (8.1) | 29 (9.8) | 38 (12.8) |
|  | Boolean | 0 | 2 (0.7) | 6 (2.0) | 11 (3.7) | 14 (4.7) | 17 (5.7) | 26 (8.8) |
| **Adalimumab-EU**  n=300 | **DAS28-4(CRP) <2.6**  Yes | 8 (2.7) | 17 (5.7) | 34 (11.3) | 48 (16.0) | 62 (20.7) | 81 (27.0) | 99 (33.0) |
|  | **ACR/EULAR remission** |  |  |  |  |  |  |  |
|  | Yes^a^ | 3 (1.0) | 5 (1.7) | 11 (3.7) | 21 (7.0) | 24 (8.0) | 44 (14.7) | 44 (14.7) |
|  | Boolean | 2 (0.7) | 3 (1.0) | 7 (2.3) | 14 (4.7) | 19 (6.3) | 32 (10.7) | 27 (9.0) |

*Adalimumab-EU* adalimumab sourced from the European Union, *ACR* American College of Rheumatology, *DAS28-4(CRP)* Disease Activity Score-28: 4 components based on high-sensitivity C-reactive protein, *EULAR* European League Against Rheumatism, *ITT* intent-to-treat

^a^Met either the Simplified Disease Activity Index or Boolean criteria for remission
