# Supplementary material for: A comparative clinical study of PF-06410293, a candidate adalimumab biosimilar, and adalimumab reference product (Humira®) in the treatment of active rheumatoid arthritis
Source: Arthritis Res Ther. 2018 Aug 15;20:178. doi: 10.1186/s13075-018-1676-y (PMC6094896; doi:10.1186/s13075-018-1676-y)
Supplement: Supplementary file 8 — All-causality treatment-emergent adverse events in at least 2% of patients in any treatment arm (safety population). (DOCX 49 kb) [file 13075_2018_1676_MOESM8_ESM.docx]

**Additional file 8** All-causality treatment-emergent adverse events in ≥2% patients in any treatment arm (safety population)

|  | **PF-06410293**  n=297  n (%) | **Adalimumab-EU**  n=299  n (%) |
| --- | --- | --- |
| **Any adverse event** | 143 (48.1) | 143 (47.8) |
| Anemia | 9 (3.0) | 2 (0.7) |
| Injection site reaction | 5 (1.7) | 6 (2.0) |
| Bronchitis | 2 (0.7) | 6 (2.0) |
| Upper respiratory tract infection | 6 (2.0) | 12 (4.0) |
| Viral upper respiratory tract infection | 21 (7.1) | 18 (6.0) |
| Alanine aminotransferase increased | 8 (2.7) | 13 (4.3) |
| Aspartate aminotransferase increased | 7 (2.4) | 7 (2.3) |
| Arthralgia | 6 (2.0) | 1 (0.3) |
| Back pain | 5 (1.7) | 7 (2.3) |
| Headache | 10 (3.4) | 8 (2.7) |
| Hypertension | 8 (2.7) | 13 (4.3) |

Includes all data collected since first injection of the study drug

*Adalimumab-EU* adalimumab sourced from the European Union
