# Supplementary material for: A comparative clinical study of PF-06410293, a candidate adalimumab biosimilar, and adalimumab reference product (Humira®) in the treatment of active rheumatoid arthritis
Source: Arthritis Res Ther. 2018 Aug 15;20:178. doi: 10.1186/s13075-018-1676-y (PMC6094896; doi:10.1186/s13075-018-1676-y)
Supplement: Supplementary file 9 — ADA and NAb incidence by study visit (safety population). Abbreviations: ADA anti-drug antibody, NAb neutralizing antibody. (DOCX 52 kb) [file 13075_2018_1676_MOESM9_ESM.docx]

**Additional file 9** ADA and NAb incidence by study visit (safety population)

| **Study visit** | **ADA and NAb status**^a^ | **PF-06410293**  n=297  n (%) | **Adalimumab-EU**  n=299  n (%) |
| --- | --- | --- | --- |
| Week 0 (Baseline) | ADA‑positive | 10 (3.4) | 11 (3.7) |
|  | NAb‑positive (% of ADA+ patients) | 8 (80.0) | 5 (45.5) |
|  | NAb‑negative (% of ADA+ patients) | 2 (20.0) | 6 (54.5) |
|  | NAb‑positive (% of total patients) | 8 (2.7) | 5 (1.7) |
|  | ADA‑negative | 287 (96.6) | 287 (96.0) |
|  | ADA not done | 0 | 1 (0.3) |
| Week 2 | ADA‑positive | 24 (8.1) | 26 (8.7) |
|  | NAb‑positive (% of ADA+ patients) | 6 (25.0) | 3 (11.5) |
|  | NAb‑negative (% of ADA+ patients) | 18 (75.0) | 23 (88.5) |
|  | NAb‑positive (% of total patients) | 6 (2.0) | 3 (1.0) |
|  | ADA‑negative | 267 (89.9) | 269 (90.0) |
|  | ADA not done | 6 (2.0) | 4 (1.3) |
| Week 6 | ADA‑positive | 40 (13.5) | 35 (11.7) |
|  | NAb‑positive (% of ADA+ patients) | 19 (47.5) | 13 (37.1) |
|  | NAb‑negative (% of ADA+ patients) | 21 (52.5) | 22 (62.9) |
|  | NAb‑positive (% of total patients) | 19 (6.4) | 13 (4.3) |
|  | ADA‑negative | 254 (85.5) | 258 (86.3) |
|  | ADA not done | 3 (1.0) | 6 (2.0) |
| Week 12 | ADA‑positive | 77 (25.9) | 91 (30.4) |
|  | NAb‑positive (% of ADA+ patients) | 31 (40.3) | 20 (22.0) |
|  | NAb‑negative (% of ADA+ patients) | 46 (59.7) | 71 (78.0) |
|  | NAb‑positive (% of total patients) | 31 (10.4) | 20 (6.7) |
|  | ADA‑negative | 214 (72.1) | 193 (64.5) |
|  | ADA not done | 6 (2.0) | 15 (5.0) |
| Week 26 | ADA‑positive | 112 (37.7) | 130 (43.5) |
|  | NAb‑positive (% of ADA+ patients) | 29 (25.9) | 30 (23.1) |
|  | NAb‑negative (% of ADA+ patients) | 83 (74.1) | 99 (76.2) |
|  | NAb‑positive (% of total patients) | 29 (9.8) | 30 (10.0) |
|  | ADA‑negative | 173 (58.2) | 142 (47.5) |
|  | ADA not done | 12 (4.0) | 27 (9.0) |
| Overall^b^ | ADA‑positive | 132 (44.4) | 151 (50.5) |
|  | NAb‑positive (% of ADA+ patients) | 41 (31.1) | 42 (27.8) |
|  | NAb‑negative (% of ADA+ patients) | 91 (68.9) | 109 (72.2) |
|  | NAb‑positive (% of total patients) | 41 (13.8) | 42 (14.0) |
|  | ADA‑negative | 165 (55.6) | 147 (49.2) |
|  | ADA not done | 0 | 1 (0.3) |

*ADA* anti-drug antibody, *adalimumab-EU* adalimumab sourced from the European Union, *NAb* neutralizing antibody

^a^ADA‑positive and -negative test results were defined as ADA titer ≥1.88 and <1.88, respectively. NAb‑positive and negative results were defined as NAb titer ≥0.70 and <0.70, respectively. Not done: Samples were not collected or collected but not analyzed. The percentage of ADA‑positive patients is based on the number of patients randomized to each treatment group. The percentage of NAb‑positive patients is based on the number of ADA‑positive patients or the total number of patients in each treatment group at the specified visit

^b”^Overall” includes data from Week 2, Week 6, Week 12, Week 26, End-of-treatment/Early Termination, Follow-up and Unplanned visits in treatment period 1
